# A Lipidomic Analysis of Leaves of Esca-Affected Grapevine Suggests a Role for Galactolipids in the Defense Response and Appearance of Foliar Symptoms

**DOI:** 10.3390/biology9090268

**Published:** 2020-09-04

**Authors:** Piebiep Goufo, Isabel Cortez

**Affiliations:** 1 Centre for the Research and Technology of Agro-Environment and Biological Sciences, Universidade de Trás-os-Montes e Alto Douro, Quinta de Prados, 5000-801 Vila Real, Portugal; icortez@utad.pt; 2 Departamento de Agronomia, Universidade de Trás-os-Montes e Alto Douro, Quinta de Prados, 5000-801 Vila Real, Portugal

**Keywords:** Esca, lipids, metabolomics, brown wood streaking, lipidome, tiger-stripes, phospholipids, sterols, mass spectrometry, hormones

## Abstract

Both qualitative and quantitative changes occur in the lipid composition of *Vitis vinifera* L. tissues, which may compromise the defense response against Esca complex disease, a widespread and damaging trunk disease. In this study, a lipidomic analysis of grapevine leaves is conducted to assess how lipid membrane remodeling relates to the emergence and progression of Esca foliar symptoms. In total, 208 molecular species (including lipids, four hormones, and some other compounds of the metabolism of lipids) were detected. Lipid species were readily assigned to the classes fatty acyls, glycerolipids, glycerophospholipids, sphingolipids, sterols, and prenol lipids. Using different clustering analyses, distinct metabolic pathways stimulated at different stages of disease development were characterized. These analyses revealed consistent changes in the abundance of 13 galactolipids and two diacylglycerolipids. Overall, the observations indicated an increment in the levels of these lipid species in leaves of asymptomatic vines and a progressive drop with increasing foliar symptom severity in symptomatic vines. Five fatty acids also appear to exert a central role in the etiopathogenesis of Esca complex disease because of their accumulation in leaves of asymptomatic vines, namely, heptadecanoic, linoleic, γ-linolenic, arachidonic, and stearic acids. Symptomatic leaves were characterized by high levels of all lipid classes, except for galactolipids, lyso-galactolipids, and compounds relevant to the biosynthesis of chlorophylls and carotenoids, that exhibited decreased levels. The data also suggested a jasmonic acid-associated signaling mechanism activation upon the invasion of woods by Esca-associated fungi, compared with abscisic and salicylic acids. Further research is required for validation of these results with additional molecular analyses using more vine cultivars.

## 1. Introduction

The plant cell represents a complex of molecules combined with numerous additional components. Plant growth and development depends on the interaction between these components and environmental stimuli, such as pathogen attacks [1,2,3,4]. Plant response to pathogens is complex and composed of at least two layers: A basal, broad-band immunity called pathogen-associated molecular pattern-triggered immunity and a strain-specific level of immunity known as effector-triggered immunity. During the establishment of these immune responses, several compounds act as signals that trigger and mediate defense responses [5,6,7,8,9]. In general, the perception and communication of mechanisms triggered by each type of immunity are facilitated via Ca^2+^ channels, mitogen-activated protein kinase (MAPK), nitric oxide (NO)-stimulated and reactive oxygen species (ROS)-regulated signaling cascades, which act as upstream sensors. This ultimately leads to the expression of defense-related genes mediated by signaling pathways dependent or independent on the hormones salicylic acid (SA), jasmonic acid (JA), and ethylene (ET), which function as secondary messengers [4,10,11,12,13,14,15]. For example, the biosynthesis of camalexin, the major phytoalexin in Arabidopsis and Brassicaceae, is triggered by the mitogen-activated protein kinases MPK3/MPK6 in an ET-independent manner, leading to resistance against *Botrytis cinerea* [13]. The perception of pathogens by plant cells also involves the generation of plant-derived signals that are specifically sensed by the host and used to direct downstream defensive cascades. These signals result from structural and biochemical changes such as (i) desaturase-mediated alterations in membrane lipid composition, and (ii) enzymatic and non-enzymatic genesis of bioactive lipids e.g., oxylipins and fatty acids (FAs) [4,16,17,18].

In several plant species, membrane lipid remodeling is a relevant strategy adopted by cells to counteract environmental stresses [1]. Specific lipid species regulate membrane fluidity, stability, and permeability during plant responses to microbial pathogens. For instance, C16 and C18 FAs contribute to the biosynthesis of the plant cuticle, which forms the first barrier against pathogens [16]. Several studies have highlighted the multifaceted roles of galactolipids in plant stress responses. The overall stability of the membrane depends on the degree of unsaturation of acyl chains of lipids and the fine orchestration of the balance between levels of the galactolipids digalactosyldiacylglycerol (DGDG) and monogalactosyldiacylglycerol (MGDG) [1,7,19,20]. The DGDG/MGDG ratio is also critical for correct protein folding and intracellular protein trafficking in the chloroplast [14,20]. Reduced photosynthesis observed in plants under stresses is a direct consequence of insufficient DGDG [14,21]. Okazaki et al. [22] have recently identified a novel galactolipid, glucuronosyldiacylglycerol, that accumulates as a protection mechanism upon phosphorus starvation.

Some lipids play very specific regulatory roles in plant defense signaling. An excellent example is oleic acid (OLE); reducing OLE levels can generate broad-spectrum disease resistance to biotrophic pathogens [10]. Sphingolipids regulate the programmed cell death (PCD) response during plant defense. Ceramide accumulation, for example, was shown to modulate PCD in Arabidopsis cells undergoing hypersensitive response [16]. Additionally, lyso-phosphatidylcholine acts as a major signal in the arbuscular mycorrhizal symbiosis between plant roots and beneficial fungi [23].

There is strong evidence that slight changes in cellular lipid composition directly or indirectly alter the coordinating events underlying abscisic acid (ABA), JA, and SA production. Reduction in OLE level in Arabidopsis induces defense responses by upregulating the expression of a variety of structurally diverse *R* genes in an SA-independent manner [10]. Phosphatidic acid (PA) binds and stimulates the ROS-generating activities of Respiratory burst oxidase homolog proteins D and F, which is important for ABA-mediated stomatal closure [4,14]. PA also stimulates MGDG synthesis by binding to *MGD1*, the major MGDG-synthesizing enzyme [14].

In some instances, the presence of pathogens at the infection site is followed by the induction of defense responses in distal undamaged tissues. These systemic responses confer long-lasting and broad-spectrum resistance throughout the plant, and include (i) induced systemic resistance (ISR), which is triggered in response to root colonization by beneficial bacteria, and (ii) systemic acquired resistance (SAR), which is activated in response to pathogen infection [4,12]. For example, specific unsaturated FA levels had a priming effect on insect elicitor-induced responses in plants, resulting in de-novo JA accumulation in unaffected organs [15]. Recent works have demonstrated an important role for galactolipids in the modulation of the JA pathway; mutations in *DGD1*, the major DGDG-synthesizing enzyme, severely reduce DGDG content and induce JA overproduction, resulting in stunted growth [21]. MGDG and DGDG also function nonredundantly to regulate SA levels and SAR [12]; specifically, DGDG is required for pathogen-responsive NO and SA accumulation at an early step during SAR, while MGDG regulates the biosynthesis of the SAR signals azelaic acid (AzA) and glycerol-3-phosphate (G3P) that function downstream of NO [12].

Studies related to the lipid composition of different tissues of grapevine plants in response to stresses are relatively recent, especially concerning pests and diseases. Moreover, these studies have largely been limited to the investigation of changes at the broad class level for a limited number of lipid species. For instance, it was shown that lipid signaling events play an important role in the resistance of grapevine to the biotrophic oomycete *Plasmopara viticola* (causal agent of downy mildew), through changes in α-linolenic acid (ALA) levels, JA biosynthesis, and phospholipase A activity in leaves [7,9]. Ceramides and derivatives of arachidonic (ARA) and eicosapentaenoic acids produced by *P. viticola* sporangia were detected in diseased tissues at the very early stages of the infection process, but not in healthy tissues [24]. Besides downy mildew, *V. vinifera* is prone to several other economically damaging diseases, such as grapevine trunk diseases (GTDs). The GTD Eutypa dieback reportedly promotes the inhibition of ALA and γ-linolenic acid (GLA) in favor of linoleic acid (LA) in asymptomatic and symptomatic leaves of affected vines [25].

Esca complex disease (ESCA) is another GTD that attacks the perennial organs of grapevines, leading to tiger-stripe symptoms on leaves, apoplexy of shoots [2,3], and extensive wood necrosis (white rot, brown streaking, and other types of dark necrotic spots) on the trunk and cordons [26] colonized by fungi, such as *Phaeomoniella chlamydospora* [5,27,28,29], *Phaeoacremonium minimum* [5,30], *Fomitiporia mediterranea* [31,32], *Botryosphaeriaceae* species, and other fungal taxa [33,34,35,36,37,38,39,40]. Protection against GTDs relies mainly on prophylactic measures, remedial surgery [26,39], and the application of biological agents [8,31] or mixtures of fertilizers [40,41] to reduce symptom incidence and severity; these control strategies often offer only partial protection. One of the main concerns in agriculture today is the reduction of pesticide use [29,31,42,43]. Consequently, identifying resistance biomarkers is vital in attempts to define new control strategies toward sustainability.

While no grapevine cultivar is known to be completely resistant to ESCA, degrees of susceptibility ranging from highly susceptible to tolerant have been reported [44]; however, the plant defense mechanisms underlying these observations have not been completely elucidated. Studies that have focused on identifying the host response to ESCA have found that the wood/foliar symptomatology described above is linked to characteristic adaptive physiological [38] and metabolic changes [36]. A prominent change observed during metabolomic profiling experiments is the presence of large amounts of stilbene phytoalexins in symptomatic tissues of ESCA-affected vines [33,34,45]. Proteomic and transcriptomic analyses have shown that the apoplexy form of ESCA induces biosynthesis of pathogenesis-related proteins (*PR*) [33], and upregulation of glutathione-S-transferase genes in green stems, cordons, and the trunk [35]. However, nothing is known concerning the lipid-associated signaling events that occur after pathogen invasion, which ultimately triggers the activation of defense responses. This knowledge remains a crucial step to better understand the infection process and appreciate the molecular mechanisms underlying symptom emergence.

The term “lipidomic” has been used to describe the branch of metabolomics [22,46,47,48,49] focused on “studying the composition, metabolism and biological role of lipids in cells at the level of molecular species” [16]. During the 2010s, several studies utilized lipidomic analyses for the elucidation of development-related questions in plants [1,16,17,18,20,22,47,48,50]. This led, for example, to the finding that epicuticular wax strengthening of the rice leaf sheath upon Brown planthopper feeding results from stimulation of the sterol biosynthetic pathway [50]. Such studies showcase the value of lipidomics in distinguishing pathways active during stress resistance.

To identify host lipid pathways associated with ESCA and potential lipid markers for new diagnosis strategies, a mass spectrometry (MS)-based platform was used to analyze the untargeted lipidome of vines grown under vineyard conditions. Comparisons were made between the lipid profiles of leaves of unaffected vines (healthy), leaves of wood-affected vines (asymptomatic), and leaves of affected vines (symptomatic) with varying degrees of damage.

## 2. Materials and Methods

### 2.1. Field Experimental Design

#### 2.1.1. Grapevine Cultivar and Vineyard Characteristics

The field study was conducted in the vineyard of Quinta de Nossa Senhora de Loudes located in the Douro winegrowing region of Portugal. The vineyard was planted in 22 longitudinal rows with *V. vinifera* L. ‘Malvasia Fina’, a traditional Portuguese white cultivar. A more detailed description of the vineyard, its soil, and prevailing climatic conditions was provided in Goufo et al. [36].

#### 2.1.2. Internal and External Inspection of Vines

Three major characteristics of ESCA are the undetermined period of latency within the vine (asymptomatic status) [3,38], the irregular annual recurrence of foliar symptoms in the vineyard [29,40], and the high variability of symptoms in both their discoloration and shape [26,36]. Considering these peculiarities of ESCA, affected vines were identified during a continuous survey period of 6 years, by assessing internal and external symptoms yearly after grape cluster harvesting. The experiment was restricted to four rows of vines trained to a bilateral cordon according to the royal-type trellis system. This corresponded to 243 vines that were numbered according to their place in the rows. Because permissions for uprooting the vines were denied, internal symptoms were assessed using wood cores. These cores consisted of 5-mm diameter and 50–100-mm length woods taken with a sterilized increment borer at ca. 20 cm above the soil surface and 10 cm below the head of the trunk. Various types of wood deterioration were observed in the trunks, which defines different groups of vines.

**Figure 1:**
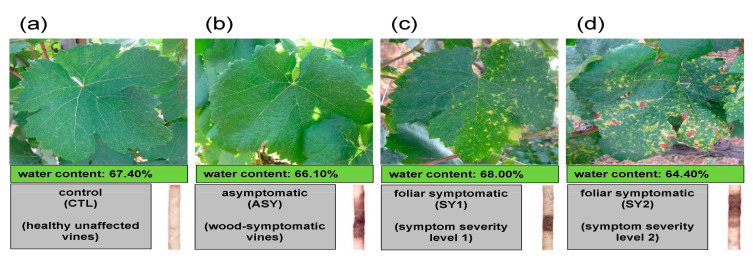
Sampling scheme: top = foliar symptom expressions of Esca complex disease (ESCA)-affected *Vitis vinifera* L. ‘Malvasia Fina’ with (**a**) healthy, (**b**) asymptomatic, (**c**) chlorotic, and (**d**) spotted/scorched leaves that will later assume a tiger-stripe pattern. Middle = water status of leaf samples estimated after oven drying. Bottom left = Experimental leaf groups: Cross-sections of some healthy trunks showed most of the tissue to be non-necrotic, whereas affected trunks showed moderate or large areas of brown wood streaks and necrotic tissues. Bottom right = cores of wood extracted with an increment borer.

Vines that did not express foliar symptoms during the study period, and showed no or minor wood deterioration uncharacteristic of ESCA [32], were selected as healthy unaffected control (CTL) vines, from which healthy leaves were sampled (Figure 1a).

Vines that displayed typical wood symptoms of ESCA, but with an absence of foliar symptoms during at least half of the 6-year survey period, including the sampling year, constituted diseased asymptomatic vines (ASY), from which healthy leaves were sampled. The deterioration observed in the wood cores could be described as a central dark brown necrosis of hard texture with sectorial black streaking of the vessels, and the vines were defined as “brown wood streaking” [36] (Figure 1b). Occasionally on some wood cores, the presence of a soft and spongy white decay was observed [33]. Because of the negligible proportion of the decay, no vine was conclusively defined as “white rot.”

The third group of vines was comprised of “brown wood streaking” vines that had continuously expressed foliar symptoms; these were considered grapevine leaf stripe disease (GLSD) symptomatic vines [40]. In the sampling year, observations were made three times per week, starting in June to record foliar symptoms as they emerged. The expansion of symptoms on leaves was monitored at the average rate of one visit per week from July through September. At harvest, symptomatic leaves were sampled on vines that first showed symptoms the same week; these leaves were arbitrarily categorized into chlorotic leaves (SY1, Figure 1c), and spotted/scorched leaves (SY2, Figure 1d), based on symptom intensity. Tiger-Striped leaves were not collected, which ensured that all analyzed leaves were in a good physiological state.

Several vines were located that had large areas of necrotic tissue, an advanced stage of degradation of peripheral tissues, and/or longitudinal cracks in the woods that could not be readily associated with ESCA. These vines were not included in the analyses, regardless of whether they expressed foliar symptoms.

#### 2.1.3. Collection and Processing of Leaf Samples

Before grape cluster harvest, six vines were sampled for each experimental group (CTL, ASY, SY1, and SY2); 10 fully developed leaves with petioles were cut with scissors from both sides of the canopy of each vine, gathered, immediately frozen with liquid nitrogen, and stored at −80 °C. Healthy and symptomatic leaves maintained a water content of ca. 65% under field conditions, although leaves that experienced more damage had the lowest water content (Figure 1a–d). Before lipidomic analyses, samples were lyophilized and ground to a fine powder in a blender.

### 2.2. Lipidomic Analyses

#### 2.2.1. Extraction of Compounds

The extraction and analysis of compounds were based on an established method [46], using the automated MicroLab Liquid Handling System (Hamilton Robotics, Inc., Reno, NV, USA). A more detailed description of the extraction, separation, and identification of compounds is provided in [51]. Briefly, 400 µL methanol were added to powdered leaves (20 mg), and the mixture was shaken vigorously for 2 min, followed by centrifugation at 1500 × *g* for 5 min at room temperature. The resulting supernatant was divided into four aliquots, which were desiccated and stored at −80 °C. Additionally, 50 mg of leaf sample was extracted with volumes of methanol/chloroform/water (2:2:1.8 *v/v/v*) based on Okazaki et al. [22]. After transesterification of the resulting extract with 14% boron trifluoride in methanol [52,53], a residue was obtained, stored at −80 °C, and constituted the fifth aliquot. Before chromatographic separation, the aliquots were defrosted overnight under nitrogen.

#### 2.2.2. Chromatographic Separations

Compounds from the five aliquots were separated on an Ultrahigh Performance Liquid Chromatography (ACQUITY UPLC, Waters Corporation, Milford, MA, USA) and a Gas Chromatography (GC-2010 Plus, Shimadzu, Kyoto, Japan) equipment. All equipment was connected to a Q-Exactive Hybrid Quadrupole-Orbitrap high resolution/accurate MS interfaced with a heated electrospray ionization source (Thermo Fisher Scientific, Waltham, MA, USA). Aliquots 1, 2, 3, and 4 were resuspended in different solutions and analyzed by UPLC under conditions optimized for the elution of hydrophilic (LC/MS Pos early), hydrophobic (LC/MS Pos late), basic (LC/MS Neg), and polar (LC/MS Polar) compounds, respectively [46]. Aliquot 5 was reconstituted in hexane and analyzed by GC under conditions optimized for the elution of free FA, as previously described [36]. All reconstituted aliquots were spiked with a series of retention time markers before injection into the MS.

#### 2.2.3. Data Processing and Identification of Lipids

Data acquisition was performed using the vendor-supplied software for the instruments [36,46]. Detected peaks with their respective intensities were extracted and organized by the mass-to-charge ratio (*m/z*), and retention index (RI). RI were calculated based on retention time markers using the Metabolon’s Laboratory Information Management System (https://www.metabolon.com). Peak annotation was conducted by comparing their specific *m/z*, fragment ion spectra, and RIs to those in the Metabolon library of ~10,000 purified standards, within user-provided ranges [46]. When isomers belonging to the same isobaric species were detected, they were denoted by adding a number sign (#) after the isobaric species name or a number within brackets after its abbreviation. In the final compound tallies, only one channel (LC/MS Polar, LC/MS Pos Late, LC/MS Pos Early, LC/MS Neg, or GC/MS) was chosen to represent compounds detected in more than one aliquot. Detected compounds are listed according to Metabolync™ (https://www.metabolon.com) sub-pathways in Table S1. Peaks were quantified as area-under-the-curve detector ion counts. The median value was calculated for each lipid species and used as the normalization factor to obtain scaled imputed data used in statistical analyses. The whole dataset with lipid masses and levels is deposited in Mendeley Data [51].

#### 2.2.4. Statistical Calculations and Data Presentation

Statistical analyses were conducted using the Microsoft Excel add-in application “Statistical Analysis Tool” [54]. Before analyses, missing values were imputed with the minimum observed value for each lipid species. Molecular species that differed between experimental groups were identified through a multiple-step procedure. Fold changes were calculated according to the ratios for log2(ASY/CTL), log2(SY1/CTL), and log2 (SY2/CTL) values. Welch’s two-sample *t*-tests were performed after log10-transformation of scaled imputed data; statistical differences were declared for *P* ≤ 0.10. An estimate of the false discovery rate (*q*-value) was also calculated at the 10% level of significance to account for multiple comparisons. Principal Components Analysis (PCA) was performed on log10-transformed and pareto-scaled data, and the first and second components were used to single out lipid species that discriminated against the experimental groups. Data are presented in Tables and graphical displays using box-and-whisker, volcano, and pie charts in Microsoft Excel 2016. For each pairwise comparison, the dataset was visualized by plotting pathway enrichment charts (rich factors) (Table S2) using bubble charts.

## 3. Results

### 3.1. Identification of Lipids Species, Hormones and Other Compounds of the Metabolism of Lipids in Grapevine Leaves

In this study, a workflow was established based on a multiplexed UPLC- and GC-MS platform to study healthy and diseased leaves of ESCA-affected vines. The conditions applied detects 208 molecular species covering a broad range of Lipid Metabolites and Pathways Strategy (LIPID MAPS) classes, including fatty acyls, glycerolipids, glycerophospholipids, sphingolipids, sterols, and prenol lipids (Table 1). Additionally, four hormones and some other compounds of the lipid metabolism were detected, as described in Goufo and Cortez [53]. Specifically, ABA, JA, and SA-glc were detected by LC/MS Neg while SA was detected by LC/MS Polar. Fifty signals were attributable to typical polar and apolar lipids, but could not be annotated. The same numbers of molecular species were found in all samples, although some compounds were present at very low levels, below the quantification limit in some ASY and SY1 samples (data not shown). Five long-chain polyunsaturated FAs (ARA/C20:4n6, ETA1/C20:3n6, ETA2/C20:3n6, EPA/C20:5n3, and DHA/C22:6n3) and two odd-chain FAs (cisPDA/C15:1n5 and cisHDA/C17:1n7) were detected and included in the analyses. Since Esca-associated fungi have never been detected in the leaves [33,37], these compounds could have been produced by other grapevine pathogens [24].

### 3.2. Control Samples versus Asymptomatic Samples

**Figure 2:**
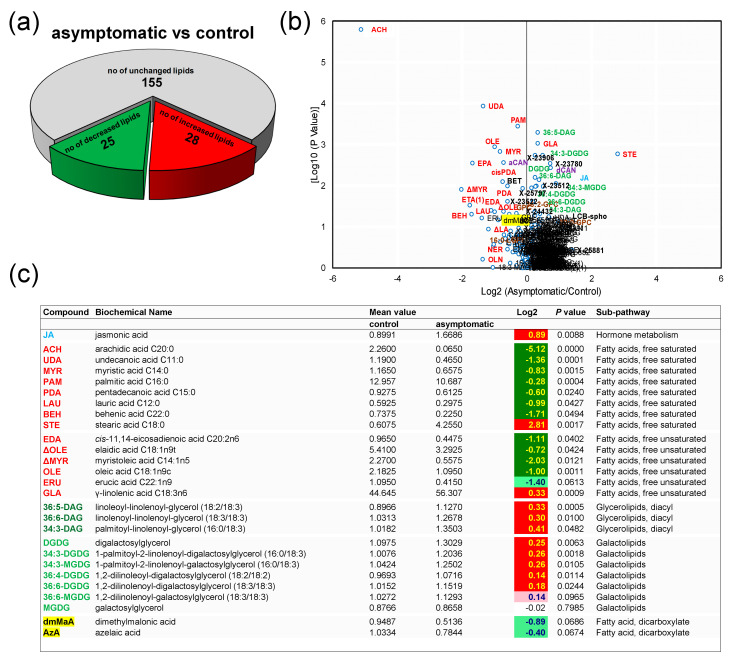
Variations in levels of hormones, lipid species, and other compounds of the metabolism of lipids in *Vitis vinifera* L. ‘Malvasia fina’ affected by Esca complex disease (ESCA): (**a**) Pie plot representing the numbers of altered species in healthy leaves from foliar-asymptomatic and wood-symptomatic vines (ASY) relative to healthy leaves from healthy unaffected control vines (CTL). Levels were considered altered when they underwent a *p*-value ≤ 0.10 (Welch’s two-sample *t*-test, *n* = 6). (**b**) Volcano plot (log2 (ASY/CTL) vs. [log10 (*p*-value)]) illustration of species differently accumulated in CTL and ASY. Abbreviations are as listed in Figure 2c and Table S1. (**c**) Representative compounds listed along a color gradient according to sub-pathways. Additional altered compounds can be found in Table S1. For log2 values, different colors represent different directions of change at *p* ≤ 0.05 (dark green = negative change; red = positive change) and at *p* ≤ 0.10 (light green = negative change; rose = positive change).

When CTL and ASY were contrasted, 53 molecular species were found to be associated with the presence of ESCA symptoms in the wood—28 were increased in ASY, whereas 25 were decreased (Figure 2a).

A volcano plot of fold change vs. log10 (*p*-value) showed that CTL and ASY exhibited distinct lipidomic profiles with clear variations among lipid species belonging to five sub-pathways (Figure 2b). ASY was associated with relatively modest 0.14- to 0.41-fold increases in the steady-state abundance of galactolipids and diacylglycerolipids (with the exception of MGDG whole level remained constant) (Figure 2c and Table S1). Although small in magnitude, these increases involving a large number of species from an abundant lipid class represent substantial changes in the total lipid content of the host cells and in the flux through biosynthetic pathways. As a general observation, ASY caused a decline in free unsaturated fatty acids (UFA) and free saturated fatty acids (SFA). The highest decrease was observed for arachidic acid (ACH, 5.12-fold change). Exceptions were GLA, stearic (STE), ARA, heptadecanoic (HAD), and tricosanoic acids. A decreasing trend in levels of dicarboxylic FAs was observed. e.g., azelaic acid (AzA, 0.40-fold decrease).

ASY was uniquely reflected in a 0.89-fold increase in the level of JA, whereas the levels of SA, ABA, and SA-glc remained relatively constant (Figure 2c and Table S1).

### 3.3. Control Samples versus Symptomatic Samples (Severity Level 1)

**Figure 3:**
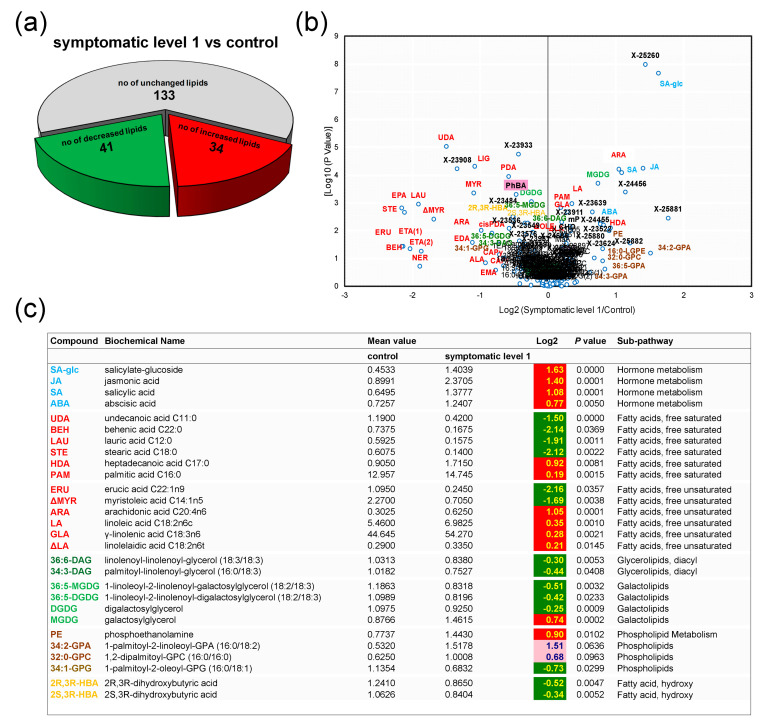
Variations in levels of hormones, lipid species, and other compounds of the metabolism of lipids in *Vitis vinifera* L. ‘Malvasia fina’ affected by Esca complex disease (ESCA): (**a**) Pie plot representing the numbers of altered species in chlorotic leaves from foliar-symptomatic and wood-symptomatic vines (SY1) relative to healthy leaves from healthy unaffected control vines (CTL). Levels were considered altered when they underwent a *p*-value ≤ 0.10 (Welch’s two-sample *t*-test, *n* = 6). (**b**) Volcano plot (log2 (SY1/CTL) vs. [log10 (*p*-value)]) illustration of species differently accumulated in CTL and SY1. Abbreviations are as listed in Figure 3c and Table S1. (**c**) Representative compounds listed along a color gradient according to sub-pathways. Additional altered compounds can be found in Table S1. For log2 values, different colors represent different directions of change at *p* ≤ 0.05 (dark green = negative change; red = positive change) and at *p* ≤ 0.10 (light green = negative change; rose = positive change).

Differences were also established between CTL and SY1: The numbers of increased and decreased molecular species after symptom emergence were 34 and 41, respectively (Figure 3a).

Compounds that exhibited the greatest change, and strongly contributed to group separation, as identified by the volcano plot (Figure 3b), are listed in Figure 3c. Symptom apparition in the leaves (SY1) was negatively associated with a range of species, including all diacylglycerolipids, all galactolipids (except for a 0.74-fold increase for MGDG), all SFAs (except for HDA and palmitic acid), the majority of hydroxy FAs, and half of the UFAs. This was especially evident for STE (2.19-fold decrease) (Figure 3c). The data also indicated that during the initial stages of symptom development, chlorophyll/carotenoid metabolism was already altered, as shown by a 0.47-fold decrease in pheophorbide A (PhBA) (Table S1). Moderate perturbation of the phospholipid metabolism was also associated with the appearance of foliar symptoms, notably an increase in phosphoethanolamine, and a decrease in 1-palmitoyl-2-oleoyl-GPG (16:0/18:1) (Figure 3c).

On the other hand, SY1 had higher levels of hormones than did CTR, i.e., 1.63-, 1.40-, 1.08-, and 0.77-fold changes in SA-glc, JA, SA, and ABA, respectively (Figure 3c).

### 3.4. Control Samples versus Symptomatic Samples (Severity Level 2)

**Figure 4:**
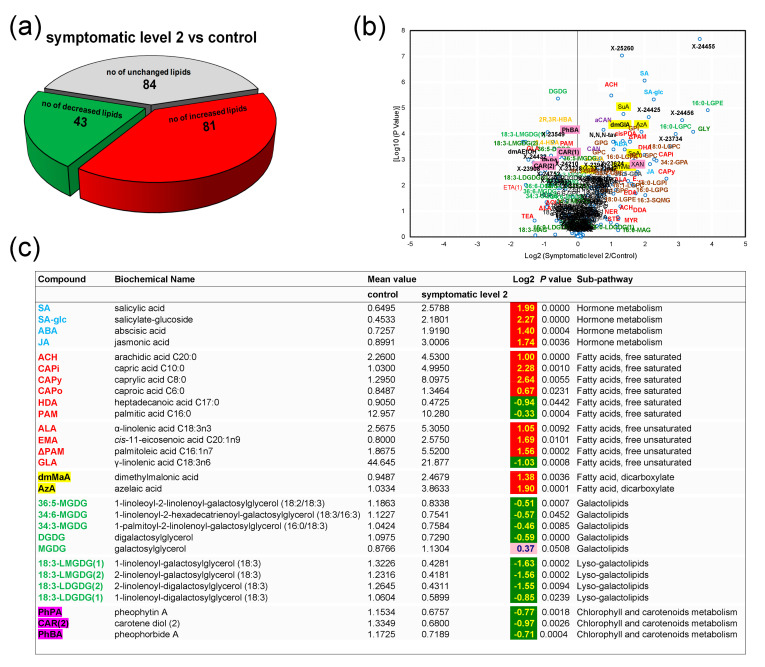
Variations in levels of hormones, lipid species, and other compounds of the metabolism of lipids in *Vitis vinifera* L. ‘Malvasia fina’ affected by Esca complex disease (ESCA): (**a**) Pie plot representing the numbers of altered species is spotted/scorched leaves from foliar-symptomatic and wood-symptomatic vines (SY2) relative to healthy leaves from healthy unaffected control vines (CTL). Levels were considered altered when they underwent a *p*-value ≤ 0.10 (Welch’s two-sample *t*-test, *n* = 6). (**b**) Volcano plot (log2 (SY2/CTL) vs. [log10 (*p*-value)]) illustration of species differently accumulated in CTL and SY2. Abbreviations are as listed in Figure 4c and Table S1. (**c**) Representative compounds listed along a color gradient according to sub-pathways. Additional altered compounds can be found in Table S1. For log2 values, different colors represent different directions of change at *p* ≤ 0.05 (dark green = negative change; red = positive change) and at *p* ≤ 0.10 (light green = negative change; rose = positive change).

A comparison of the lipid profiles of CTR and SY2 was also performed and showed that with symptom extension on the leaves, the number of molecular species with increased levels rose (from 34 in SY1 to 81 in SY2), whereas the number of molecular species with decreased levels remained unchanged (from 41 in SY1 to 43 in SY2) (Figure 4a).

A volcano plot (Figure 4b) indicated that hormones, lipid species, and other compounds altered with foliar symptom progression were spread across 16 sub-pathways. This included increased levels of all phospholipids (except for a 0.32-fold decrease in 1,2-dilinolenoyl-GPC (18:3/18:3)), lyso-phospholipids, amide, and amino FAs, monoacylglycerolipids, metabolites of the carnitine metabolism (Table S1), dicarboxylic Fas, and hormones (Figure 4c). The highest increases were recorded for several lyso-phospholipids, e.g., a 3.88-fold increase in 1-palmitoyl-sn-glycero-3-phosphoethanolamine (16:0-LGPE). Similar increases were seen with several hydroxy FAs, sulfolipids, metabolites of the choline metabolism (Table S1), UFAs, and SFAs (Figure 4c). On the other hand, levels of all galactolipids (except for MGDG having a 0.37-fold increase) and lyso-galactolipids and of the majority of metabolites of the chlorophyll/carotenoid metabolism were depleted in diseased leaves (Figure 4c and Table S1). The levels of galactolipids varied based on the extent of leaf damage, and the greater the damage, the larger the decreases. For example, chlorosis emergence on leaves (SY1) led to a 22% decrease in DGDG, which was further decreased by 108% with the appearance of scorching and spotting (SY2).

### 3.5. PCA Highlight of Holistic Differences among Experimental Groups

**Figure 5:**
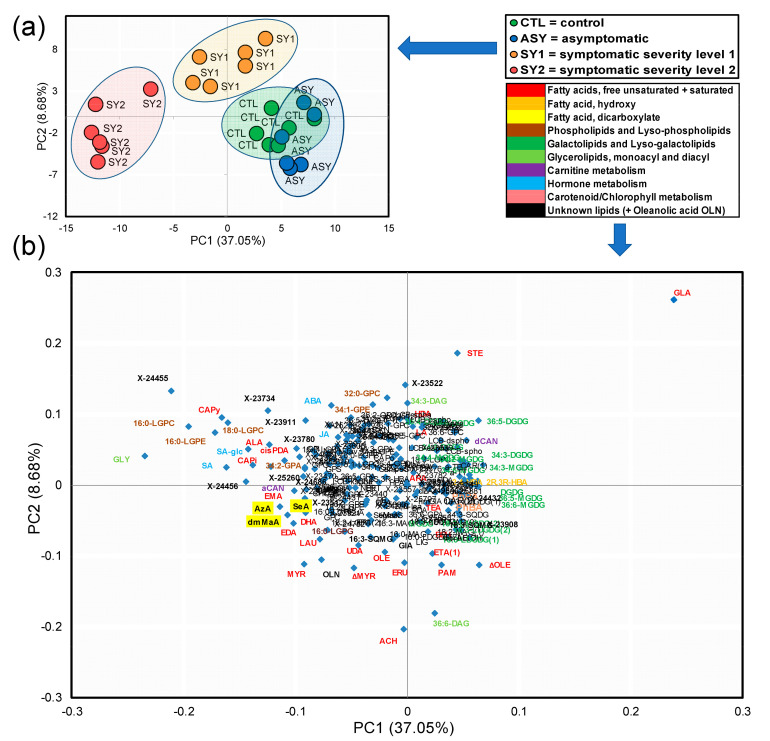
Principal component analysis (PCA) of lipidome data (lipid species, hormones, and other compounds of the metabolism of lipids) of leaves of *Vitis vinifera* L. ‘Malvasia Fina’ affected by Esca complex disease (ESCA). (**a**) Loading plot of the first two principal components explaining 45.73% of total data variance. Four-Leaf groups were separated, as shown in the legend on the right. (**b**) Score scatter plot showing lipid species that strongly contributed to group separation. Abbreviations correspond to compounds in Table S1.

The ability of differentially displayed molecular species to segregate CTR, ASY, SY1, and SY2 was represented by PCA plots (Figure 5a,b).

The score plot showed that samples were clustered with respect to their corresponding leaf groups (Figure 5a), suggesting that each group had a distinct lipidome profile, although there was a partial overlap between CTL and ASY. The most discriminating compounds were identified on the loading plot, based on high and low eigenvalue scores on PC1 and PC2 (Figure 5b).

### 3.6. Identification of Top Lipid Candidate Markers

**Figure 6:**
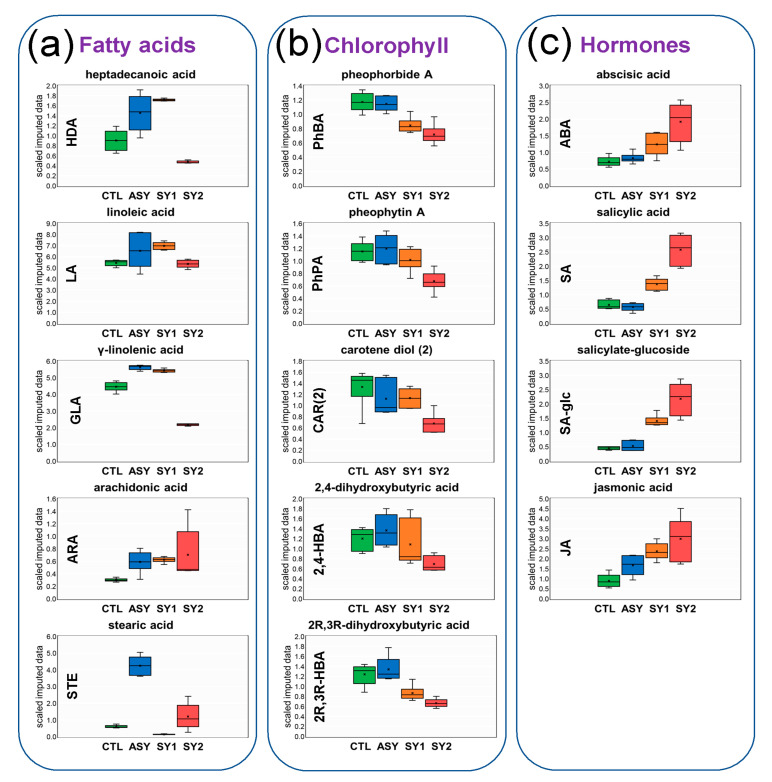
Box-And-Whisker plots corresponding to hormones, lipid species, and other compounds of the metabolism of lipids with specific and consistent changes in response to Esca complex disease (ESCA) in *Vitis vinifera* L. ‘Malvasia Fina’ leaves. Scaled imputed data were calculated by median value normalization of area-under-the-curve detector ion counts for each compound (names on the top and abbreviations on the *Y*-axis). The rounder corner shapes delimit a cluster of individuals of similar sub-pathways: (**a**) Free FAs, (**b**) hydroxy FAs and metabolites of chlorophyll/carotenoid metabolism, and (**c**) hormones. CTL = healthy leaves from healthy unaffected control vines, ASY = healthy leaves from foliar-asymptomatic and wood-symptomatic vines, SY1 = chlorotic leaves from foliar-symptomatic and wood-symptomatic vines, SY2 = spotted/scorched leaves from foliar-symptomatic and wood-symptomatic vines. High levels of ARA probably produced by grapevine pathogens were found and the compound included in the analyses.

**Figure 7:**
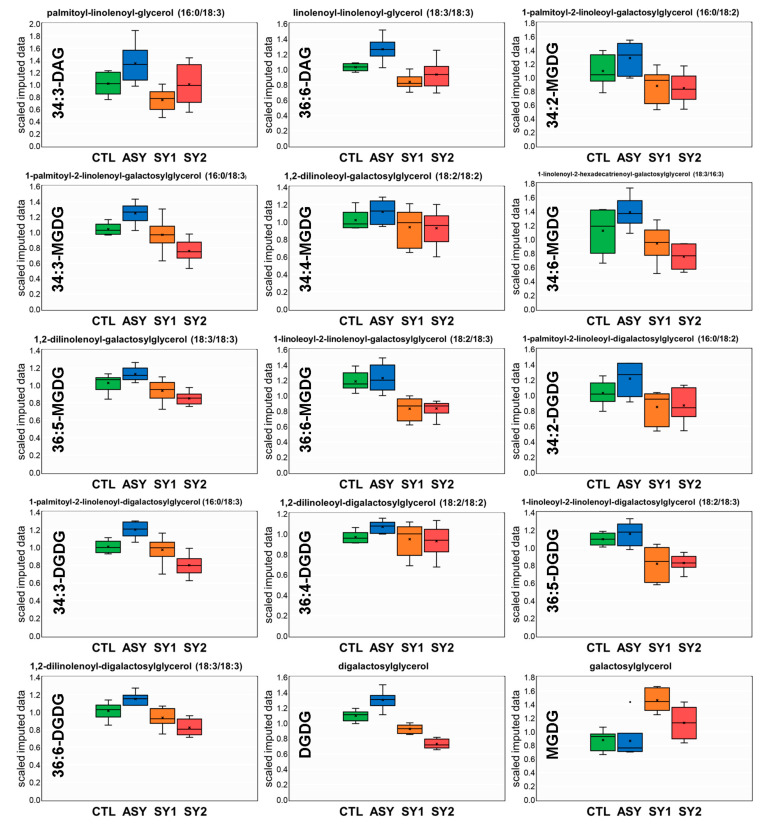
Galactolipid and diacylglycerolipid profiles (names on the top and abbreviations on the *Y*-axis) of leaves of Vitis vinifera L. ‘Malvasia Fina’ affected by Esca complex disease (ESCA). Levels of individual lipid species are expressed as scaled imputed data, as detailed in the Materials and Methods Section. CTL = healthy leaves from healthy unaffected control vines, ASY = healthy leaves from foliar-asymptomatic and wood-symptomatic vines, SY1 = chlorotic leaves from foliar-symptomatic and wood-symptomatic vines, SY2 = spotted/scorched leaves from foliar-symptomatic and wood-symptomatic vines.

Biomarkers of symptom emergence and disease progression were selected based on insights gained from the analysis of fold changes, Welch’s two-sample *t*-test data, volcano plots, and PCA. These combinations of techniques illustrated that a few lipid species discriminate leaves of ESCA-affected vines from leaves of healthy vines (Figure 6 and Figure 7).

The separation of the different leaf groups along PC2 (Figure 5b) resulted from lipid species with high-fold increases in the SY2/CTR comparison (+X, −Y axes), e.g., 16:0-LGPE (3.88-fold change), and those with moderate-fold increases in the SY2/CTR comparison, fold decreases in the SY1/CTR, and ASY/CTR comparisons (−X, −Y axes), e.g., ACH (1.00-, 0.98-, and 5.12-fold changes). Included were the hormones ABA, SA, SA-glc, and JA, whose levels increased or tended to increase with disease progression and foliar symptom severity (Figure 6c).

While the intensities of several FAs changed in ASY, SY1, and SY2 relative to CTL, specific responses were recorded for HAD, LA, GLA, ARA, and STE, which were the only FAs loading on PC1 (+X, +Y axes) (Figure 5b). With few exceptions, levels of these FAs tended to increase in ASY and SY1, and decrease in SY2 (Figure 6a).

Lipid species with high-fold decreases in all the comparisons loaded on PC1 (+X, −*Y*-axis), e.g., elaidic acid (ΔOLE, 0.72, 0.22, and 0.91-fold decreases). Other compounds that showed a relatively constant pattern of change and loaded on PC1 (+X, −Y axes) were the hydroxy FAs 2,4-dihydroxybutyric (2,4-HBA) and 2R,3R-dihydroxybutyric (2R,3R-HBA) acids, the prenol lipid carotene diol, and the compounds PhBA and pheophytin A (PhPA) (Figure 5b). With disease progression, levels of these compounds appeared to evolve from the basal composition in ASY to less than that of CTL levels in SY1 and SY2 (Figure 6b).

At the lipid class level, two of the most evident changes across all of the pairwise comparisons were increased levels of 12 galactolipids and two diacylglycerolipids during the latency period of the disease, and a decrease with increasing symptom severity. These compounds all loaded on PC1, forming an arc cloud in Figure 5b. Box-and-whisker plots illustrating levels of these lipid species are shown in Figure 7, which demonstrates the evolution of lipid remodeling during ESCA progression.

The different classification techniques also select the 13 top unidentified lipid candidates (Table 2). Several of these molecular species showed a strong accumulation in both SY1 and SY2. This was particularly true for X-24455 and X-24456; whole levels also tended to increase with symptom progression from the wood to the leaves. X-23908 represented an exception with depleted levels in SY1 and SY2. Efforts to identify and functionally characterize these additional species are ongoing.

### 3.7. Pathway Enrichment Analysis of Differentially Accumulated Lipid Species

**Figure 8:**
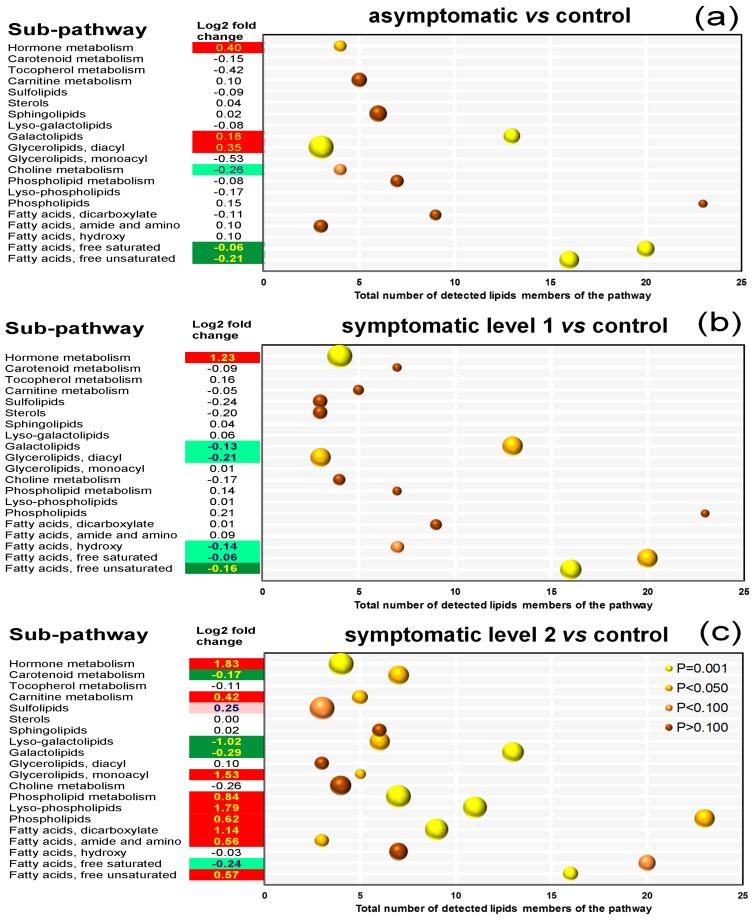
Rich factor analysis of the differentially accumulated molecular species in leaves of *Vitis vinifera* L. ‘Malvasia Fina’ affected by Esca complex disease (ESCA). The comparisons were made between (**a**) healthy leaves from healthy unaffected control vines (CTL) and healthy leaves from foliar-asymptomatic and wood-symptomatic vines (ASY), (**b**) CTL and chlorotic leaves from foliar-symptomatic and wood-symptomatic vines (SY1), and (**c**) CTL and spotted/scorched leaves from foliar-symptomatic and wood-symptomatic vines (SY2). The involved sub-pathways (*Y*-axis) are plotted based on levels of 158 annotated compounds. The *P*-value (Welch’s two-sample *t*-test) and rich factors are represented by the color and the size of the bubbles, respectively. The rich factor indicates the ratio of the number of altered molecular species in a lipid sub-pathway relative to the total number of identified species belonging to the sub-pathway (*X*-axis). Log2-fold changes were calculated using the sum of scaled imputed mean values of all compounds in a sub-pathway.

Besides the statistical methods described above, an additional line of evidence (Figure 8a–c) was taken into consideration when interpreting lipidomic changes observed as the result of ESCA.

This consisted of examining unaffected lipid species and their inclusion in a common pathway with top biomarkers. A rich factor analysis contributed to sorting different compounds according to their sub-pathways (Table S2). The projections in Figure 8a,b demonstrate that the top seven modulated sub-pathways rated by *P*-values and rich factors (bubble size) in the ASY/CTL and SY1/CTL comparisons were relevant to the metabolism of JA, the biosynthesis of diacylglycerolipids, UFAs, galactolipids, SFAs, hydroxy FAs, and the metabolism of choline. For the SY2/CTL comparison, 14 highly enriched sub-pathways were identified (Figure 8c), including five of the sub-pathways in Figure 8a,b (hydroxy FAs and choline were not affected). Although levels of lipid species of choline metabolism tended to decrease in ASY, a meaningful change was observed for only betaine (0.60-fold decrease). Hydroxy FAs, on the other hand, were mainly depleted in biological processes related to the progression of foliar symptoms (Table S1). Hence, these sub-pathways are unlikely to play pivotal roles in the infection process.

## 4. Discussion

Membrane lipids play structural, regulatory, and antimicrobial roles in the adaptation and survival of plants subjected to stressful conditions. The application of lipidomic approaches in some abiotic stress studies, such as phosphorus deficiency [22], nitrogen starvation [17], drought [18,20,47], and low and high temperatures [1,47], has contributed considerably to unraveling these roles. Such an approach was used in the present study to provide characteristic lipid signatures and mechanistic insights to disease progression in leaves of grapevines affected by ESCA (Figure 1).

### 4.1. Mobile Signals Are Generated in the Fungi-Infected Woods and Systemically Activate a Defense Response in Healthy Leaves

Literature data indicate that ESCA is a multi-factor disease, and many complex pathological scenarios could explain the emergence of symptoms. GLSD, characterized by tiger stripe-like symptoms on the leaves, is the most common expression of the disease [3,44]. ESCA-associated fungi are undetected in the distal organs of the vines (i.e., annual stems and leaves), demonstrating that vascular occlusions and symptoms occur at a distance from the pathogen niche localized in the wood [2]. Exhaustive research has shown that losses in grape yield and quality caused by ESCA are correlated with leaf symptom severity [34,40]. These observations led to the hypothesis that leaf studies could generate new perspectives regarding (i) the nature and cause of ESCA symptoms, and (ii) the applicability of leaf candidate markers for predicting the onset of these symptoms.

In this study, changes in the levels of several FAs in ASY versus CTL were observed (Figure 2). This included reductions in the levels of the majority of SFAs and UFAs, e.g., OLE and ΔOLE (C18:1). These findings, confirmed by different independent statistical approaches, are consistent with several reports on FA alterations associated with plant diseases [7,10,15,50]. However, a major point makes these results novel and unique, i.e., the fact that these changes were observed in healthy leaves from asymptomatic vines as opposed to diseased leaves. This indicated that some signals are transported from the site of local infection in the trunk to remote parts of the vine.

SAR is a mechanism of induced broad-spectrum resistance against secondary infections in the whole plant, following localized infection by a pathogen [12]. In plants, FAs and their derivatives have been described as distal signaling molecules in very few biological processes, mainly in wounding and in interactions with insect herbivores [15]. In this study, data are presented that show that systemic changes in FA flux also occurred in distal organs of the grapevine, which is important for SAR. Several chemical inducers of SAR have been identified, which are transported a remote distance in plants and include SA, methylSA, pipecolic acid, dehydroabietinal, G3P, and AzA [4]. SA, G3P, and AzA were identified in this study (Figure 2 and Table S1); the first two compounds remained unaffected, whereas AzA levels decreased in ASY relative to that of CTL. AzA is a dicarboxylic FA that is generated by oxidative cleavage of C18 UFAs that contain a double bond at carbon 9, such as OLE, LA, ALA, and GLA. AzA induces SAR via its effect on G3P biosynthesis [4]. The repression of AzA biosynthesis in ASY and SY1 does not support its involvement in SAR.

The C18 FAs that serve as precursors for AzA are mainly derived from MGDG and DGDG [12,19,21]. Increases in levels of galactolipids in ASY (except for MGDG) was a major finding of this study (Figure 7), concomitant to commensurate increments in levels of STE (C18:0), LA (C18:2, *P* > 0.10), GLA (C18:3), and ARA (C20:4) (Figure 6a). It is possible that a threshold level of these galactolipids and FAs is necessary to generate AzA in sufficient quantity to induce SAR. A report by Gao et al. [12] supports this fact, given that both MGDG and DGDG at specific concentrations were able to generate AzA and its precursor, 9-oxononanoic acid, when exposed to superoxide radical under in vitro conditions. DGDG is notably known for its requirement for pathogen-induced SA biosynthesis during SAR [1,4]. Colonization of woods by ESCA-associated fungi probably generates an unknown signal that, together with DGDG, triggers independent signaling events that lead to the accumulation of other galactolipids, but not SA. DGDG is also known to acquire fluidity to the membranes [7,19,20]. Consequently, accumulation of DGDG, and not an increase of the unsaturation degree, in ASY may be the cellular mechanism controlling the structural stability of membranes under latency, which may be crucial in the prevention of symptom appearance.

### 4.2. Except for Galactolipids and Prenol Lipids, There Is a Positive Association between Lipid Levels and Symptom Severity

With symptom emergence and progression, an increment was observed in the levels of most lipids. The number of FAs whose levels increased in grapevine leaves rose with increasing leaf damage, from 5 in ASY to 7 in SY1 and 10 in SY2. Levels of some specific FAs were consistently elevated in diseased leaves, including LA, ΔLA, and GLA in SY1 (with symptom progression, the levels reverted), OLE, ALA, and ARA in SY2 (Figure 3, Figure 4, Figure 5 and Figure 6 and Table S1). Although for ΔOLE and STE, the pattern of change appeared diffused (Table S1), these data reinforce the important role C18 FAs play as ubiquitous molecules recruited in multiple biotic and abiotic pathways [4]. Increased lipid unsaturation has long been considered one of the most striking features of stress-tolerant plants [18,22,47]. The trend for a higher unsaturation grade of FAs in SY1 and SY2 may help to counteract the oxidative burst occurring with symptom progression. Progressive accumulation of phospholipids and lyso-phospholipids was also observed with increasing symptom severity (Figure 3 and Table S1). Specific lipids of phospholipid metabolism, such as PA, are associated with both host-pathogen interactions and abiotic stress responses [1,16,18,29].

Accumulation of galactolipids in tissues has been correlated with enhanced disease tolerance in many plant species [4,47]. Upon *P. viticola* pathogen challenge, for example, levels of MGDG and DGDG increased in leaves of the resistant *V. vinifera* ‘Regent’, whereas no change was recorded in those of the susceptible Trincadeira [7]. That pattern of change did not hold for ESCA-affected vines: Levels of all galactolipids and diacylglycerolipids fell with increasing leaf damage, except for that of MGDG, which rose (Figure 7). Distinct responses for MGDG and DGGD might be explained by the fact that the galactolipid synthases *MGD1* and *DGD1* which catalyze consecutive galactosyltransfer reactions, localize to the inner and outer chloroplast envelopes, respectively, necessitating intermembrane lipid transfer [14]. Literature data suggest that MGDG and DGDG are required at distinct steps and function exclusively in their individual roles during the induction of SAR and plant defenses [12,14]. MGDG is essential for thylakoid biogenesis in plant leaves and contributes to the rigidity of membranes. Remarkably, MGDG depletion in tissues has been reported under heat stress [1], drought stress [18], and nitrogen deficiency [17]. However, the catabolic process of MGDG was partly attributed to transcriptional downregulation of genes involved in the prokaryotic pathway and instability of FA desaturase proteins [1]. MGDG changes in ESCA-diseased leaves should theoretically lead to a more rigid membrane, which may physically delay leaf damage.

It was also observed that the levels of some hydroxy FAs (e.g., 2,4-HBA, 2R,3R-HBA) and compounds involved in the biosynthesis of chlorophylls and carotenoids (e.g., PhBA, PhPA) decreased with symptom emergence and progression (Figure 6b). The data compare well with those of Zhang et al. [50], who observed that Brown planthopper infestation caused chlorophyll degradation in rice leaves. Hydroxy FAs are important constituents of the cutin, with which the waxes form the cuticle, the first line of contact with the environment [4,16]. The drop in hydroxy FAs levels indicated increased permeability of the cuticle, and hence, the improbability that increases in levels of MGDG, UFAs, and phospholipids described above was sufficient to reinforce wax ester layers as a physical barrier to avoid leaf damage.

### 4.3. Galactolipids Likely Play a Role in the Mechanism Underlying Inhibition of Symptom Formation

It is known that besides pathogens, multiple factors, such as climatic/edaphic conditions and cultural practices, contribute to ESCA development [3,26,32,38,39]. Physiologically, the apparition of foliar visual symptoms has been attributed to two main events: (i) The production of tyloses and gels by the vine, which together with the fungi occlude the vessels (hydraulic failure hypothesis), and/or (ii) the production of plant-derived signals and pathogen molecules in the transpiration stream, which is translocated through the phloem and accumulated in the canopy (elicitor-toxin hypothesis), both leading to leaf desiccation [2,3,37]. Despite the actual physiological events, ESCA pathogenesis in Malvasia Fina appears to involve changes in levels of a whole lipid class, namely, galactolipids, linked to delaying (ASY), triggering (SY1), and/or accelerating leaf damage (SY2) (Figure 7). This was evident when lipid species were assigned to common metabolic pathways, and galactolipid biosynthesis was found to be highlighted in all the pairwise comparisons in pathway enrichment analyses (Figure 8 and Table S2).

Diagnosis of ESCA is not easy because of the time delay between wood infection and symptom expression, the unpredictable year-to-year discontinuity in symptom expression, and the differential disease manifestations [29,41,44]. The present criteria for ESCA diagnosis consist of uprooting or cutting the vines or the invasive collection of wood samples, all of which are costly. An early biochemical marker of fungal invasion or a prognostic indicator of the presence of the disease in asymptomatic vines would be extremely helpful in establishing the most adapted treatment aimed at limiting grape losses and vine death. Efforts have recently been made to discover leaf biochemicals that could assist in the current diagnostic approaches or could predict the evolution of ESCA. Some researchers have observed that some amino acids, polyphenols, and energy metabolism-relevant molecules, undergo enormous changes in asymptomatic leaves [33,34,35,36,44], but there is no clear link between these alterations and foliar symptom emergence. In this study, very good correlations were found between the level of leaf necrosis and levels of all 13 identified galactolipids (not shown). Together, these observations suggest an intimate connection between galactolipid homeostasis and foliar expression of ESCA symptoms, and these lipid species may be suitable targets for early diagnosis of the disease.

### 4.4. Accumulation of JA in All Stressed Leaves Suggests a Signaling Role in Grapevine Defense Response to ESCA

The enhanced coverage of grapevine lipids achieved in this study (Table 1) observed differentially regulated lipids, which belonged principally to pathways related to the biosynthesis of UFAs, SFAs, diacylglycerolipids, galactolipids, and hormones (Figure 8). Systemic examination of FA levels showed no consistent alteration of lipids belonging to the same class, i.e., UFAs or SFAs (Figure 2, Figure 3 and Figure 4), suggesting these pathways might not be easily amenable to intervention, except for the C18s. The biological relevance of alterations observed for diacylglycerolipids remains to be ascertained with more structured studies, given that only a handful (three) of them were detected (Figure 2 and Figure 3).

The most obvious changes in lipid metabolism were presented by the galactolipids (discussed above) and the hormone JA. A link between galactolipids and JA biosynthesis was recently uncovered; mutations in *DGD1* led to reduced DGDG content and induced JA overproduction caused by an increased MGDG/DGDG ratio [21]. In this study, JA accumulation was common for all leaf groups (ASY, SY1, and SY2) despite different changes in MGDG and DGDG levels (Figure 6c), allowing hypotheses for its direct contribution to intercellular communication and grapevine tolerance to ESCA. In response to pathogen invasion, the synthesis of signal transduction mediators, such as JA is transiently increased to activate downstream signaling pathways, leading to physiological responses [9,11,15]. The biosynthesis of JA occurs via the hexadecanoid (derived from 16:3) and/or octadecanoid (derived from 18:3) pathways, depending on the plant species [11,15]. In grapevine, JA synthesis via the octadecanoid pathway seems to be predominant [11]. Although the relationship between JA levels and genes and enzymes of the JA metabolic network was not explored in this study, from the literature review, there is evidence to suggest that the involved reactions might be differentially regulated upon ESCA affection. Accumulation of JA in leaves, as well as upregulation of genes associated with its biosynthesis and activation, have recently been found as supporting the response of grapevine to *P*. *viticola* challenge [7,9]. JA-mediated signaling has been associated with plant defense against necrotrophic pathogens and insects, inducing the accumulation of secondary metabolites and *PR*s; increased levels of phytoalexins and *PR*s is a common response of grapevine tissues to ESCA-associated fungi [5,33,35,45].

Plant resistance to biotrophic and hemibiotrophic pathogens is believed to be mediated through SA signaling, leading to hypersensitive response and SAR [12]. SA is a small phenolic compound synthesized via the shikimate pathway. DGDG is also known to contribute to SA biosynthesis. Despite increased levels of DGDG, data from this study did not agree with an early upregulation of SA in response to wood invasion by ESCA-related fungi. However, with symptom emergence, it appears that synergistic interactions between SA and JA pathways allowed the vine to finely-tune its defense responses (Figure 6c).

A typical reaction of grapevine tissues to drought is the biosynthesis of ABA, which regulates transpiration-mediated stomatal closure [4,41]. Consistent with the results of this study (Figure 6c), elevated levels of ABA have been detected in tiger-striped leaves [3]. However, in black-streaked woods of GLSD and apoplectic vines, ABA is barely accumulated [33], suggesting that the appearance of foliar symptoms cannot be simply considered a water-deficit-induced alteration. Promotion of SA and ABA accumulation in diseased leaves of ESCA-affected vines could reflect a specific perturbation of metabolism caused by the impairment of the conducting vessels. These hormones would be required to maximize the defense output, but with a beneficial effect only during the early stages of symptom appearance.

## 5. Conclusions

The lipidomic data reported in this study suggested that bulk reprogramming of lipid metabolism in response to the invasion of vine wood by ESCA-associated fungi could contribute to suppression of foliar symptom manifestation, as attested by increased levels of galactolipids. These changes probably helped to maintain membrane integrity and normal protein function during the latency period, in addition to stimulating the downstream pathways leading to the activation of the host’s defenses. However, two major limitations of the study were the low sample size, i.e., only one grapevine cultivar was used, and the disadvantage linked to using wood cores for selecting asymptomatic vines. These limitations should be added to the fact that the study was conducted under natural vineyard settings. Whether or not lipidomic changes observed were environment-specific or mainly held information about the particular cultivar-pathogen interaction is unknown, though the results reflected real field conditions. The next logical step to this study is to follow up with asymptomatic vines during disease development and compare the lipid signatures of leaves of as many cultivars as possible under different environments. A prerequisite to these additional studies is to agree on a precise and standardized definition of “asymptomatic” and “symptomatic” vines.

## Figures and Tables

**Table 1:** Lipid classes, metabolic pathways, employed chromatography platform/ionization polarity, and a number of molecular species profiled in the study.

LIPID MAPS Class ^1^	Sub-Pathway ^2^	KEGG Map ^2^	Detection Platform ^3^	No of Species
Fatty acyls	Fatty acids, free saturated	01212	LC/MS Neg, GC/MS	16
Fatty acyls	Fatty acids, free unsaturated	01040	GC/MS	20
Fatty acyls	Fatty acids, hydroxy	00073	LC/MS Polar	7
Fatty acyls	Fatty acids, amide and amino	00061	LC/MS Pos Early, LC/MS Pos Late	3
Fatty acyls	Fatty acids, dicarboxylate	00071	LC/MS Polar	9
NA	Carnitine metabolism	00260	LC/MS Pos Early	5
NA	Hormone metabolism	04075	LC/MS Neg, LC/MS Polar	4
Glycerolipids	Glycerolipids, monoacyl	00561	LC/MS Neg	5
Glycerolipids	Glycerolipids, diacyl	00561	LC/MS Pos Late	3
Glycerophospholipids	Galactolipids	00564	LC/MS Pos Late	13
Glycerophospholipids	Lyso-galactolipids	00564	LC/MS Pos Late, LC/MS Neg	6
Glycerophospholipids	Sulfolipids	00564	LC/MS Neg	3
Glycerophospholipids	Phospholipids	00564	LC/MS Pos Late	23
Glycerophospholipids	Lyso-phospholipids	00564	LC/MS Pos Late	11
NA	Phospholipid metabolism	00564	LC/MS Pos Early, LC/MS Polar	7
NA	Choline metabolism	00564	LC/MS Pos Early	4
Sphingolipids	Sphingolipids	00600	LC/MS Pos Late	6
Sterol lipids	Sterols	00100	LC/MS Pos Late	3
Prenol lipids	Tocopherol metabolism	00130	LC/MS Pos Late	3
NA	Carotenoid/Chlorophyll metabol	00860/00906	LC/MS Pos Late, LC/MS Neg	7
Unknown lipids	NOT APPLICABLE (NA)	NA	LC/MS Neg, LC/MS Pos Early	50

^1^ Lipids were classified according to the Lipid Metabolites and Pathways Strategy (LIPID MAPS, https://www.lipidmaps.org). ^2^ Pathways were classified according to Metabolon Pathway Orders (Metabolync™, https://www.metabolon.com) and the Kyoto Encyclopedia of Genes and Genomes (KEGG, https://www.genome.jp/kegg/). ^3^ Five different chromatographic conditions were used in the Lipidomic platform and are described in the “Materials and Methods” Section.

**Table 2:** Characteristics of 13 unidentified lipids species that strongly discriminated leaf groups of *Vitis vinifera* L. ‘Malvasia Fina’ affected by ESCA. White color = *p* > 0.10 for unaffected levels; dark green = *p* ≤ 0.05 for decreased levels, light green = *p* ≤ 0.10 for decreased levels; red = *p* ≤ 0.05 for increased levels; and rose = *p* ≤ 0.10 for increased levels (Welch’s two-sample *t*-test).

Unknown Lipid	Detection Platform	Retention Indice (RI)	Molecular Ion	ASY/CTL	SY1/CTL	SY2/CTL
X-24686	LC/MS Pos Early	1876	224.1126	−0.05	**0.25**	**0.62**
X-25260	LC/MS Neg	1465	315.0721	−0.07	**1.43**	**1.32**
X-24455	LC/MS Pos Early	2641	237.0867	0.20	**0.82**	**3.64**
X-24456	LC/MS Pos Early	2538	237.0866	0.17	**1.14**	**3.11**
X-24425	LC/MS Pos Early	2378	130.0974	−0.20	−0.19	**2.12**
X-23734	LC/MS Pos Early	881	174.0758	**0.42**	0.39	**2.77**
X-23512	LC/MS Neg	1306	267.1084	**0.37**	**0.27**	**0.29**
X-23522	LC/MS Neg	1417	267.1085	**0.50**	**0.77**	**0.41**
X-23780	LC/MS Pos Early	2190	144.1017	**0.72**	0.04	−0.18
X-23911	LC/MS Neg	800	283.1037	**0.25**	**0.20**	**−0.14**
X-25880	LC/MS Pos Early	2208	222.0761	−0.12	**0.33**	0.17
X-24432	LC/MS Pos Early	618	210.0606	**−0.55**	0.07	**−1.32**
X-23908	LC/MS Neg	1237	188.0929	−0.12	**−1.34**	**−1.11**

CTL = healthy leaves from healthy unaffected control vines, ASY = healthy leaves from foliar-asymptomatic and wood-symptomatic vines, SY1 = chlorotic leaves from foliar-symptomatic and wood-symptomatic vines, SY2 = spotted/scorched leaves from foliar-symptomatic and wood-symptomatic vines.

## Supplementary Materials

The following are available online at https://www.mdpi.com/2079-7737/9/9/268/s1, Table S1: Changes in lipid levels in leaves of grapevine affected by Esca complex disease. Table S2: Alterations in lipid sub-pathways in leaves of grapevine affected by Esca complex disease.

## Abbreviations

CTL	healthy leaves from healthy unaffected control vines
ASY	healthy leaves from foliar-asymptomatic and wood-symptomatic vines
SY1	chlorotic leaves from foliar-symptomatic and wood-symptomatic vines
SY2	spotted/scorched leaves from foliar-symptomatic and wood-symptomatic vines
FA	fatty acids
DGDG	digalactosyldiacylglycerol
MGDG	monogalactosyldiacylglycerol
MAPK	mitogen-activated protein kinase
NO	nitric oxide
ROS	reactive oxygen species
PA	phosphatidic acid
OLE	oleic acid
SA	salicylic acid
JA	jasmonic acid
ET	ethylene
PCD	programmed cell death
ALA	α-linolenic acid
ARA	arachidonic acid
GTD	grapevine trunk disease
GLA	γ-linolenic acid
LA	linoleic acid
ESCA	Esca complex disease
*PRs*	pathogenesis-related proteins
MS:	mass spectrometry
GLSD	grapevine leaf stripe disease
UPLC	ultrahigh performance liquid chromatography
GC	gas chromatography
RI	retention index
PCA	principal components analysis
UFA	free unsaturated fatty acids
SFA	free saturated fatty acids
ACH	arachidic acid
STE	stearic acid
HDA	heptadecanoic acid
AzA	azelaic acid
ABA	abscisic acid
PhBA	pheophorbide A
16:0-LGPE	1-palmitoyl-*sn*-glycero-3-phosphoethanolamine
2,4-HBA	2,4-dihydroxybutyric acid
2R,3R-HBA	2R,3R-dihydroxybutyric acid
PhPA	pheophytin A
ΔOLE	elaidic acid
SAR	systemic acquired resistance
G3P	glycerol 3-phosphate
